# *Acinetobacter junii*: an emerging One Health pathogen

**DOI:** 10.1128/msphere.00162-24

**Published:** 2024-04-12

**Authors:** Alejandro Aguilar-Vera, Elena Bello-López, Gabriel Iván Pantoja-Nuñez, Gloria M. Rodríguez-López, Vladimir Morales-Erasto, Santiago Castillo-Ramírez

**Affiliations:** 1 Programa de Genómica Evolutiva, Centro de Ciencias Genómicas, Universidad Nacional Autónoma de México, Cuernavaca, México; 2 Pecuarius Laboratorios S.A. de C.V., Ciudad Obregón, Sonora, México; 3 Departamento de Microbiología e Inmunología, Facultad de Medicina Veterinaria y Zootecnia, Universidad Nacional Autónoma de México, Ciudad de México, México; E O Lawrence Berkeley National Laboratory, Berkeley, California, USA

**Keywords:** emerging pathogens, antibiotic resistance, resistome, genomic epidemiology, One Health, *Acinetobacter junii*

## Abstract

*Acinetobacter junii* is an opportunistic human and animal pathogen severely understudied. Here, we conducted the largest genomic epidemiological study on this pathogen to date. Our data show that this bacterium has spread globally. Also, we found that some human and non-human isolates are not well differentiated from one another, implying transmission between clinical and non-clinical, non-human settings. Remarkably, human but also some non-human isolates have clinically important antibiotic resistance genes, and some of these genes are located in plasmids. Given these results, we put forward that *A. junii* should be considered an emerging One Health problem. In this regard, future molecular epidemiological studies about this species will go beyond human isolates and will consider animal-, plant-, and water-associated environments.

**IMPORTANCE:**

*Acinetobacter baumannii* is the most well-known species from the genus *Acinetobacter*. However, other much less studied *Acinetobacter* species could be important opportunistic pathogens of animals, plants and humans. Here, we conducted the largest genomic epidemiological study of *A. junii*, which has been described as a source not only of human but also of animal infections. Our analyses show that this bacterium has spread globally and that, in some instances, human and non-human isolates are not well differentiated. Remarkably, some non-human isolates have important antibiotic resistance genes against important antibiotics used in human medicine. Based on our results, we propose that this pathogen must be considered an issue not only for humans but also for veterinary medicine.

## OBSERVATION

Antimicrobial drug resistance is a global health concern. The genus *Acinetobacter* has some important species causing multi-drug and even pan-drug-resistant infections, which are very difficult to treat (1). *Acinetobacter baumannii* is the most studied species from the genus. However, other species also cause human and animal infections (1). Yet, many of them have been neglected, *A. junii* being the perfect example. For instance, as of 18 August 2023, the search query “*Acinetobacter junii*” produced just 168 publications on the National Center for Biotechnology Information. Nonetheless, several reports have shown it can be an opportunistic human pathogen (2–6). Some studies suggest it can cause bacteremia or ocular infections in humans (3, 4, 6). Yet, other studies indicated it can also cause infections in animals (7–9). For instance, *A. junii* has been associated with bovine mastitis and has also been found in cattle feedlots (7, 9). Importantly, genome sequencing has been instrumental in inferring the epidemiology and the genetic basis of antimicrobial drug resistance in several *Acinetobacter* species (10–12). Lately, independent studies have published several tens of *A. junii* genomes. Although important, these studies have focused on a few isolates from just one source without taking into account all the other genomes published. No study has tried to use all this information to establish the relatedness between the human (clinical) and the non-human (animal and else) isolates. Thus, we conducted the most comprehensive genomic epidemiology study about this pathogen using all the publicly available genomes (as of 8 August 2023).

**Fig 1:**
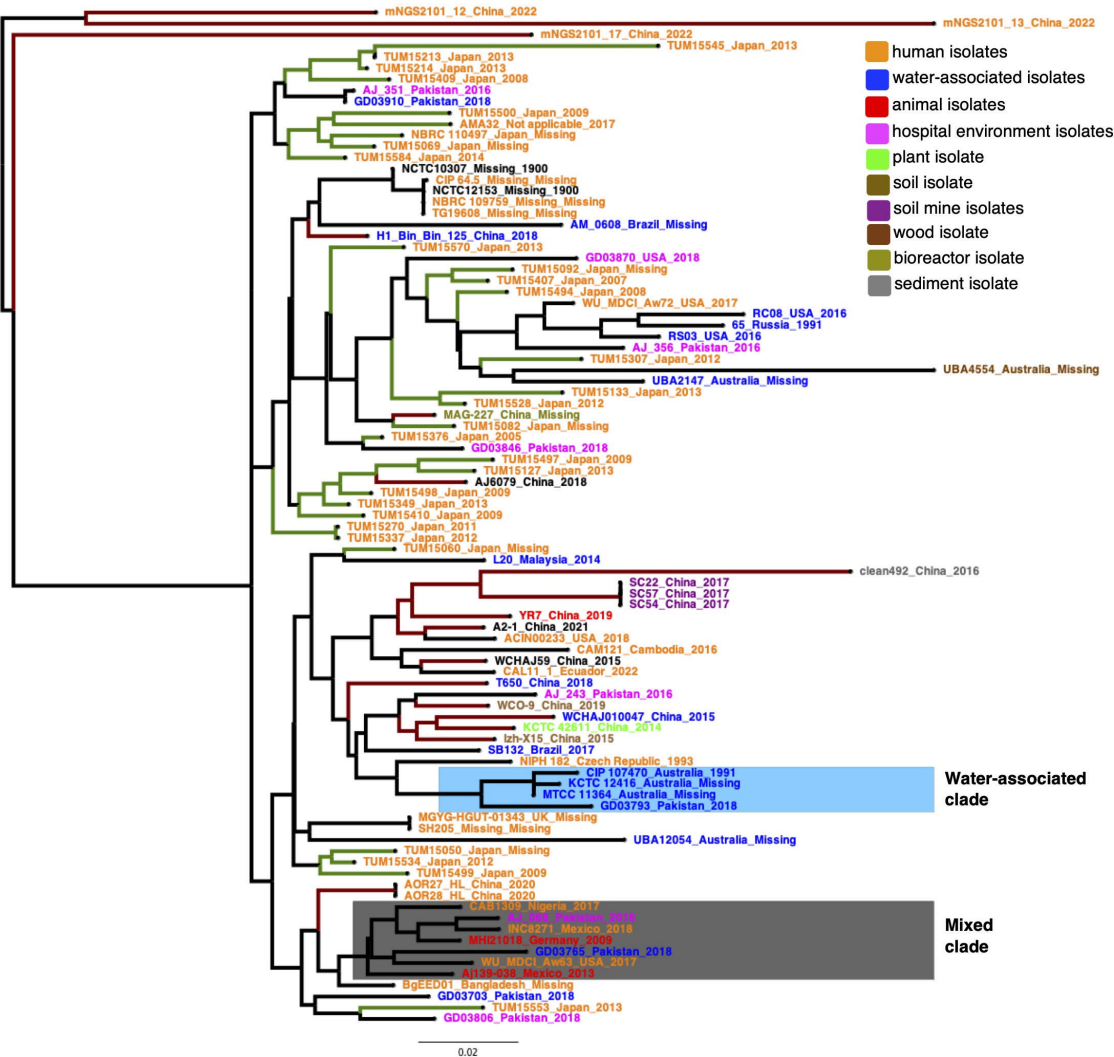
Global phylogeny. Maximum likelihood phylogeny based on the single-gene families without recombination. We used the GTR+I model to run the phylogeny. Isolates are color-coded (see color code key). The gray rectangle shows an example where human and non-human isolates are closely related, whereas the blue rectangle shows a well-differentiated clade of water-associated isolates. The green branches denote isolates/clades from Japan, whereas the dark red branches highlight isolates/clades from China. The scale bar denotes the substitutions per site.

Table S1 describes the publicly available genomes (90 isolates) employed in this study. We also included a bovine milk isolate (Aj139-038) collected from a farm in Cajeme, Sonora, Mexico, in September 2013. This isolate was sequenced at Novogene (Sacramento, CA, USA), employing a NovaSeq 6000 platform with a 150-bp paired-end configuration. The genome was assembled with SPAdes v.3.13.1 (13), annotated via PROKKA v.1.12 (14), and corroborated for completeness and no contamination using CheckM v.1.1.3 (15), as we did for *A. baumannii* (12). Our data set has 91 isolates and covers 15 countries from different continents and 31 years (see Fig. S1), being the most extensive data set ever created for this bacterium. Figure S1 shows that this bacterium has spread globally. To analyze if human and non-human isolates are well differentiated from one another, we constructed a core genome phylogeny, as we did before for *A. baumannii* (16). We created a super alignment with all the single families without recombination, and on that alignment, a maximum likelihood phylogeny was run using RAxML (17). The phylogeny was color-coded according to the host/source of the isolates (see Fig. 1). Notably, we observed that different non-human isolates do not form clear-cut major clades (groups), different from the human ones (isolates in orange). For instance, neither the water-associated isolates (blue isolates) nor the animal ones (red isolates) form single clades containing all the isolates. Nonetheless, we noted some examples where non-human isolates are differentiated from human ones. For instance, one group of water-associated isolates (light blue rectangle, Fig. 1) mainly from Australia forms a well-defined clade. There is also a group of three Chinese isolates from a soil mine (purple isolates). Contrary to that, we found a clade (gray rectangle, Fig. 1) where human isolates clustered not only with animal isolates but also with a water-associated isolate and even an isolate from a hospital environment. From a public health point of view, this implies that there is transmission between humans and animals and even water-associated settings. Geographically speaking, we noted that, within the same country, there are isolates with different genetic identities. For instance, if one looks at isolates from China (dark red branches) and Japan (green branches), countries with many isolates, one can appreciate that isolates are scattered across the tree. The same applies to other countries with fewer isolates such as Australia, Pakistan, or the USA. Thus, within some countries, several lineages are co-circulating. Collectively, these results suggest that non-human isolates are not sharply differentiated from human isolates, and there is even a case where animal, human, and water isolates are closely related. Additionally, this bacterium is globally spread, and several lineages can be found in some countries.

**Fig 2:**
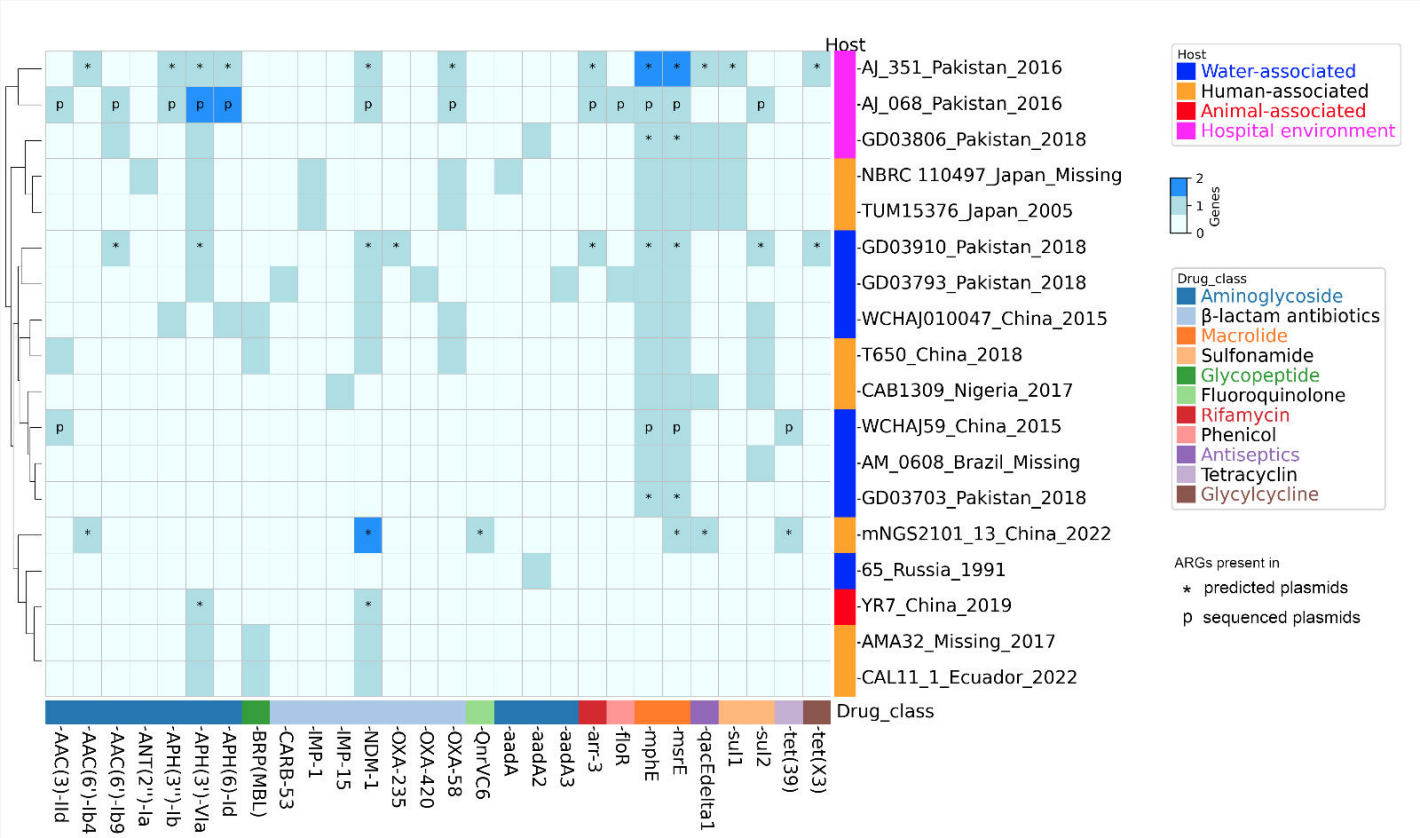
Antibiotic resistance genes. Heat map showing the antibiotic resistance genes. The *in silico* prediction of the resistome for each genome was conducted using CARD. Plasmids were predicted with MOB-suite, but only circular plasmids or incomplete but having a relaxase were considered for counting the ARGs in plasmids. These cases are denoted with an asterisk. When possible, plasmids were also considered from complete genomes; these are highlighted with the letter “p*.*” Isolate names are provided to the right. Drug classes are color-coded and at the bottom.

Concerning antibiotic resistance, many human and animal isolates seem to be susceptible to antibiotics. A clear example is Aj139-038, which was susceptible to all the 16 antibiotics tested (see File S1). Nonetheless, some human and animal isolates have important antibiotic-resistant phenotypes. For instance, colistin-resistant isolates have been described in human infections (6, 18), whereas carbapenem-resistant isolates have been reported in animals (8, 9). To better understand the genetic determinants of antibiotic resistance, we conducted an *in silico* prediction of the resistome for all the isolates (Fig. 2). We employed the Resistance Gene Identifier tool from CARD (19) using the same settings as we did before (12). Just 20% of the isolates (18 out of 91) have antibiotic resistance genes (ARGs). The two isolates with the most ARGs were AJ_068 and AJ_351. Both isolates were sampled from washroom sinks in hospital intensive care units in Pakistan. Of note, several non-human isolates carry the very important beta-lactamase gene, NDM-1. For instance, two remanent water isolates, GD03793 and GD03910, collected in 2018 from Pakistan have this gene. Also, the isolate YR7 sampled from feces in a chicken farm in China in 2019 presents this beta-lactamase gene. Finally, the sewage isolate WCHAJ010047 from China has this gene and another carbapenemase, namely, OXA-58. We also noted the presence of two other important carbapenemases (IMP-15 and IMP-1) in three human isolates from Japan and Nigeria. Plasmids disseminate ARGs; thus, we conducted an *in silico* prediction of plasmids, via MOB-suite (20), and analyzed the complete genomes for plasmids, then counted ARGs in the plasmids (see Fig. 2 and Table S2). Seven isolates had all their ARGs in plasmids, and another one had two out of seven ARGs in plasmids; these eight isolates were from different sources (humans, animals, water, and hospital environments). Of note, carbapenemases were in plasmids: NDM-1 (five isolates), OXA-58 (two isolates), and OXA-235 (one isolate). Taken together, these analyses show that not only human but also some non-human isolates have clinically relevant ARGs, and some ARGs are located in plasmids.

Our study has limitations. First, we carried out convenience sampling (gathering publicly available genomes), implying the sampling criteria were not uniform across the initial studies. Second, geographically speaking, this data set is uneven as many countries were not sampled. However, despite these limitations, this study is the most extensive ever and provides the first big picture of the genomic epidemiology of this pathogen. This study will be a reference point for more elaborate studies in the future.

In conclusion, we show that this bacterium has disseminated globally. Human and non-human isolates are not well differentiated from one another. Importantly, some non-human isolates have important antibiotic resistance genes. Thus, we assert that *A. junii* must be considered a One Health issue. Our results imply that similar to *A. baumannii* (21, 22), much more attention should be paid to the non-human, non-clinical sources of *A. junii*. In this regard, as one of us (S.C.-R.) has posited before, “We should be aiming for a global, multi-host genomic epidemiology” (23).

## Data Availability

All the genomes used in this study are listed in Table S1. This table provides the National Center for Biotechnology Information (NCBI) RefSeq assembly numbers for all of them. The newly sequenced genome was submitted to the NCBI and has NCBI RefSeq assembly number GCF_031461105.1 (BioSample SAMN37185567).

## SUPPLEMENTAL MATERIAL

File S1 msphere.00162-24-s0001.pdf Antimicrobial susceptibility testing for the isolate Aj139-038.

Figure S1 msphere.00162-24-s0002.pdf Temporal and geographic spread.

Legends msphere.00162-24-s0003.docx Captions for all the supplemental files.

Table S1 msphere.00162-24-s0004.xlsx List of the genomes employed in this study and their metadata.

Table S2 msphere.00162-24-s0005.pdf Antibiotic resistance genes present in plasmids.

ASM does not own the copyrights to Supplemental Material that may be linked to, or accessed through, an article. The authors have granted ASM a non-exclusive, world-wide license to publish the Supplemental Material files. Please contact the corresponding author directly for reuse.
