# Supplementary material for: Acinetobacter junii: an emerging One Health pathogen
Source: mSphere. 2024 Apr 12;9(5):e00162-24. doi: 10.1128/msphere.00162-24 (PMC11237400; doi:10.1128/msphere.00162-24)
Supplement: File S1 — Antimicrobial susceptibility testing for the isolate Aj139-038. [file msphere.00162-24-s0001.pdf]

Supplementary File 1

Strain	Isolation source	Geographic location	Collection date	The strain was sensitive to:	AMR genotype
Aj139-038	Bovine milk	Cajeme, Sonora, Mexico	September, 2013	PIP, TZP, TIM, SAM, CTX, CRO, CAZ, FEP, MEM, IPM, AN, GM, TET, CIP, LVX, SXT	No resistance genes were detected by CARD and ResFinder

Antimicrobial susceptibility testing was performed using the agar disk diffusion method as described in the Clinical and Laboratory Standards Institute M100-Ed32 (CLSI, 2022) for 16 antibiotics. **PIP**: Piperacillin, **SAM**: Ampicillin/Sulbactam, **TZP**: Piperacillin/Tazobactam, **TIM**: Ticarcillin/Clavulanic acid, **CAZ**: Ceftazidime, **FEP**: Cefepime, **CTX**: Cefotaxime, **CRO**: Ceftriaxone, **IPM**: Imipenem, **MEM**: Meropenem, **GM**: Gentamicin, **AN**: Amikacin, **TE**: Tetracycline, **CIP**: Ciprofloxacin, **LVX**: Levofloxacin, **SXT**: Sulfamethoxazole/ Trimethoprim.
