# Supplementary figures and images for: Acinetobacter junii: an emerging One Health pathogen
Source: mSphere. 2024 Apr 12;9(5):e00162-24. doi: 10.1128/msphere.00162-24 (PMC11237400; doi:10.1128/msphere.00162-24)

A

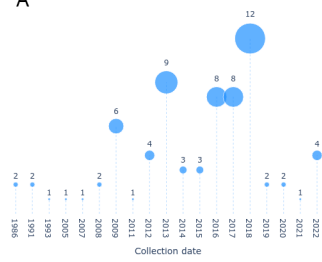

B

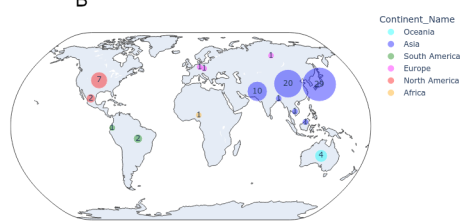

Supplement: Figure S1 — Temporal and geographic spread. [file msphere.00162-24-s0002.pdf]
