# Supplementary material for: Acinetobacter junii: an emerging One Health pathogen
Source: mSphere. 2024 Apr 12;9(5):e00162-24. doi: 10.1128/msphere.00162-24 (PMC11237400; doi:10.1128/msphere.00162-24)
Supplement: Legends — Captions for all the supplemental files. [file msphere.00162-24-s0003.docx]

**SUPPLEMENTARY MATERIAL LEGENDS**

**Supplementary Figure 1- Temporal and geographic spread**

The collection dates for the isolates that have such information (A). The geographic location of the isolates (B).

**Supplementary File 1**

Antimicrobial susceptibility testing for the isolate Aj139-038.

**Supplementary Table 1**

List of the genomes employed in this study and their metadata.

**Supplementary Table 2**

ARGs present in plasmids.
