# Supplementary material for: Acinetobacter junii: an emerging One Health pathogen
Source: mSphere. 2024 Apr 12;9(5):e00162-24. doi: 10.1128/msphere.00162-24 (PMC11237400; doi:10.1128/msphere.00162-24)
Supplement: Table S2 — Antibiotic resistance genes present in plasmids. [file msphere.00162-24-s0005.pdf]

Supplementary Table 2. ARGs present in plasmids.

Sample id	Strain	Assembly level	*ARGs	Host	Isolation source	Country	Collection date	&ARGs in predicted plasmid with relaxase or circular status or sequenced			
								plasmids	#	%	Genes
GCF_003939335.2	AJ_351	Complete Genome	14	Hospital environment	Washroom sink in hospital intensive care unit	Pakistan	2016	1	14	100	tet(X3), APH(3")-Ib, APH(6)-Id, msrE (2), mphE (2), **NDM-1**, arr-3, AAC(6')-Ib4, qacEdelta1, sul1, APH(3')-Via, **OXA-58**
GCF_003939355.2	AJ_068	Complete Genome	14	Hospital environment	Washroom sink in hospital intensive care unit	Pakistan	2016	1	14	100	**OXA-58**, msrE, mphE, APH(6)-Id (2), AAC(3)-Iid, APH(3')-Via (2), AAC(6')-Ib9, arr-3, **NDM-1**, sul2, APH(3")-Ib, floR
GCF_029841885.1	GD03806	Contig	7	Hospital environment	Surface swab	Pakistan	2018	1	2	29	mphE, msrE
GCF_000775815.1	NBRC 110497	Contig	9	Human associated	Human sputum	Japan	-	0	0	0	
GCF_009001395.1	TUM15376	Contig	7	Human associated	Human blood	Japan	2005	0	0	0	
GCF_029839935.1	GD03910	Contig	9	Water associated	Remnant water	Pakistan	2018	1	9	100	APH(3')-Via, **OXA-235**, tet(X3), sul2, msrE, mphE, **NDM-1**, arr-3, AAC(6')-Ib9
GCF_029842155.1	GD03793	Contig	8	Water associated	Remnant water	Pakistan	2018	0	0	0	
GCF_004296305.1	WCHAJ010047	Contig	8	Water associated	Sewage	China	2015	0	0	0	
GCF_021384305.1	T650	Contig	7	Human associated	Hospital ICU	China	2018	0	0	0	
GCF_030056035.1	CAB1309	Scaffold	5	Human associated	Blood culture	Nigeria	2017	0	0	0	
GCF_002165245.3	WCHAJ59	Complete Genome	4	Water associated	Sewage	China	2015	1	4	100	AAC(3)-Iid, tet(39), mphE, msrE
GCA_900299105.1	AM_0608	Contig	3	Water associated	Amazon river Upstream section	Brazil	-	0	0	0	
GCF_029843865.1	GD03703	Contig	2	Water associated	Clean catch water	Pakistan	2018	1	2	100	mphE, msrE
GCA_028614545.1	mNGS2101_13	Contig	7	Human associated	-	China	2022	1	7	100	**NDM-1 (2)**, msrE, tet(39), QnrVC6, AAC(6')-Ib4, qacEdelta1
GCF_001941805.1	65	Complete Genome	1	Water associated	Limnetic water	Russia	1991	0	0	0	
GCF_018336855.1	YR7	Complete Genome	2	Animal associated	Feces from chicken farm	China	2019	1	2	100	**NDM-1**, APH(3')-VIa
GCF_009645695.1	AMA32	Scaffold	3	Human associated	Human	-	2017	0	0	0	
GCF_030016975.1	CAL11_1	Contig	3	Human associated	Blood	Ecuador	2022	0	0	0	

* Antibiotic resistance genes (ARGs)

& Some other ARGs were in the predicted plasmids as per MOB-recon. However, to consider only reliable cases, in addition to the plasmids from complete sequenced genomes, just MOB-suite predicted plasmids with relaxases or circular status were taken into account for counting the ARGs.
